# Integrating technologies for comparing 3D gene expression domains in the developing chick limb

**DOI:** 10.1016/j.ydbio.2008.01.031

**Published:** 2008-05-01

**Authors:** Malcolm E. Fisher, Allyson K. Clelland, Andrew Bain, Richard A. Baldock, Paula Murphy, Helen Downie, Cheryll Tickle, Duncan R. Davidson, Richard A. Buckland

**Affiliations:** a Department of Cell and Developmental Biology, University of Dundee, Dow Street, Dundee, UK; b UK MRC Human Genetics Unit, Western General Hospital, Crewe Road, Edinburgh, UK; c School of Natural Science, Trinity College, College Green, Dublin 2, Ireland

**Keywords:** 3D atlas, Gene expression, Limb development, Chick embryo

## Abstract

Chick embryos are good models for vertebrate development due to their accessibility and manipulability. Recent large increases in available genomic data from both whole genome sequencing and EST projects provide opportunities for identifying many new developmentally important chicken genes. Traditional methods of documenting when and where specific genes are expressed in embryos using wholemount and section *in-situ* hybridisation do not readily allow appreciation of 3-dimensional (3D) patterns of expression, but this can be accomplished by the recently developed microscopy technique, Optical Projection Tomography (OPT). Here we show that OPT data on the developing chick wing from different labs can be reliably integrated into a common database, that OPT is efficient in capturing 3D gene expression domains and that such domains can be meaningfully compared. Novel protocols are used to compare 3D expression domains of 7 genes known to be involved in chick wing development. This reveals previously unappreciated relationships and demonstrates the potential, using modern genomic resources, for building a large scale 3D atlas of gene expression. Such an atlas could be extended to include other types of data, such as fate maps, and the approach is also more generally applicable to embryos, organs and tissues.

## Introduction

The ease of access to the embryo and subsequent manipulability has made the chick a reliable and powerful system for developmental biology. The power of this system has been enhanced recently by the availability of genomic data from both whole genome sequencing (2004) and large-scale EST projects (Boardman et al., 2002; Carre et al., 2006; Hubbard et al., 2005; Kim et al., 2006). This opens up new opportunities for identifying all the genes that mediate the development of the embryo and its constituent parts and then using high throughput methods to test their function (Brown et al., 2003). One of the first steps in this process is to document where and when specific genes are expressed in the embryo and a large amount of gene expression data is being generated. A repository of chick embryo gene expression data, GEISHA, has already been pioneered by Antin and colleagues (Bell et al., 2004). This consists of a collection of photographs of embryos in which gene expression has been assayed mainly by using whole mount *in-situ* hybridisation, in some cases accompanied by sectioned material. The database that we describe here is different in that gene expression is visualised in 3D using OPT and expression patterns are mapped onto 3D digitised images. Here we describe how we have started to establish such a database of 3D gene expression patterns for the developing chick wing and investigated some of the practicalities involved.

OPT was developed at the MRC Human Genetics Unit in Edinburgh and is one of a number of new microscopy techniques that have been developed in the last few years that allow capture of 3D image data. OPT has already been used to study the development of human, mouse, fly and plant embryos (DeLaurier et al., 2006; Kerwin et al., 2004; Lee et al., 2006; McGurk et al., 2007; Sharpe et al., 2002) and one of its advantages is that it captures the three dimensional distribution of gene expression in an intact embryo.

In order to compare large numbers of gene expression patterns, a number of recent atlas projects have taken the approach of mapping gene expression data to digital reference models. For example, the Edinburgh Mouse Atlas of Gene Expression (EMAGE) deals with 2D data in this way (Baldock et al., 2003), section *in-situ* derived gene expression data in the mouse brain have been mapped to 3D models generated by Micromagnetic resonance imaging (Lein et al., 2007) and a Zebrafish 3D anatomical Atlas has been produced based on sectioned material for the projection of gene expression data (Verbeek et al., 1999). Projects such as EMAGE (Baldock et al., 2003) and GENEPAINT (Visel et al., 2004) have begun to build large queryable databases containing both whole mount and section *in-situ* data for mouse embryos. Since the system has already been set up for mouse embryos, the establishment of a parallel database for the chick should allow direct comparisons between gene expression patterns in the two organisms. We have adopted the Bookstein thin plate spine algorithm for mapping our 3D data, which has previously been used extensively in morphometric analysis (Albertson and Kocher, 2001; Bruner et al., 2004; Harmon, 2007; Yeh, 2002). Once gene expression data are assembled in a digital atlas, powerful modern data mining techniques can be used to examine relationships potentially leading to unexpected discoveries.

We have focussed on the developing chick wing bud. The wing bud is a good test system for investigating the power of a 3D database because it is a structure with no significant morphological features at early stages. Many insights into the mechanisms that pattern the vertebrate limb have come from studies on chick embryos (Tickle, 2004). In the long term, the ability to compare multiple patterns of gene expression should enable us to identify synexpression domains, complementary patterns and possibly also discrete boundaries of gene expression. We have optimised protocols to maximise consistency of initial whole mount *in-situ* hybridisation, OPT capture and mapping data to a reference model. In this paper, we have studied a number of previously described genes in terms of their expression patterns. This has allowed us to perform a pairwise analysis of overlap of expression for an initial set of genes and to identify some previously unappreciated features of expression with respect to dorso-ventral distribution. We have also used computational techniques based on microarray analysis to look for specific regions of the limb where genes are co-expressed.

## Materials and methods

### Embryo preparation

White Leghorn chick eggs were incubated in a humidified incubator at 38 °C for the appropriate time for the desired developmental stage as determined by the Hamburger and Hamilton stages (Hamburger and Hamilton, 1951). Eggs were then windowed, embryos removed to ice-cold Phosphate Buffered Saline (PBS) (0.02 M phosphate, 0.15 M NaCl) and cleaned of extra-embryonic membranes. Eyes and forebrain were punctured with a tungsten needle to reduce trapping, and embryos were transferred to 4% (w/v) ice-cold paraformaldehyde (PFA) overnight. The embryos were then put through a graded methanol series at 4 °C; ending in 2, 100% methanol washes and stored at − 20 °C until use.

### Plasmid preparation and probe synthesis

The plasmids used for the different genes were *Shh* (Echelard et al., 1993), *Fgf8* (Crossley et al., 1996), *Msx1* (Hill et al., 1989), and *Tbx3* (Isaac et al., 1998). All plasmids were linearised and transcribed according to their sources. EST clones acquired from ARK genomics were used as probes for *Wnt3a* (EST clone 603102629F1), *Wnt5a* (EST clone 603799237F1), *Lmx1* (EST clone 603127966F1) and *HoxD13* (EST clone 603499362F1). All EST clones were in pBluescript II KS+, which was linearised with Not1 (NEB) and transcribed with T3 RNA polymerase (Roche) to produce antisense probes. Plasmids were grown up using standard protocols and purified using Qiagen plasmid mini kits and individual clones were sequenced to check their identity.

The RNA probes were synthesised accordance with standard protocols (Maniatis et al., 1982; Nieto et al., 1996) and purified using the ProbeQuant G-50 spin column system (Amersham Biosciences). In some cases probe purification was performed using phenol chloroform extraction and Lithium Chloride precipitation as detailed in Nieto et al. (1996).

### *In-situ* hybridisation

The *in-situ* protocol used was a modified version of that of Nieto et al. (1996), full details of the modified protocol are in supplementary materials.

Before scanning under UV, embryos require some further washes to remove excess NBT–BCIP. Embryos were washed twice for 10 min in PBS at RT. Embryos were then moved to 10× TBST and allowed to equilibrate at RT, this should take between 10 and 20 min depending on the size of the embryo. Embryos were then washed 3 times for 20 min in 1× TBST and left to wash overnight in fresh 1× TBST at 4 °C. Embryos were washed 3 times for 5 min in PBT at RT and then fixed overnight in 4%PFA–DEPC–PBS at 4 °C. Embryos were washed briefly a further 2 times in PBS and then refixed in formal saline.

### Section *in-situ* hybridisation

Section *in-situs* were performed on HH22 embryonic limbs according to the method of Moorman et al. (2001). The *Wnt5a* probe was the same as above.

### Tyramide Signal Amplification (TSA)

To modify the *in-situ* protocol for the fluorescent TSA colour reaction, glutaraldehyde was removed in the fixation step to minimise autofluorescence. Secondary detection was performed using a peroxidase (POD) linked anti-DIG antibody. The colour reaction was performed according to the Alexa Fluor 568 kit manufacturer’s instructions (Invitrogen — T-20914) with volumes increased to accommodate whole embryos.

### OPT sample preparation and scanning

Standard reference embryos were fixed in 4% PFA/0.2% glutaraldehyde mix, which produces a stronger autofluorescent signal than PFA alone. The addition of 0.2% glutaraldehyde to the fix was not necessary for embryos that had been *in-situ* hydbridised, due to the presence of glutaraldehyde in the fixative steps of that protocol. Reference embryos were stored in 100% methanol until scanning, at which point they were taken back through a methanol series to PBS and briefly to water. Embryos having undergone *in-situ* hybridisation were washed 3 times for 20 min in PBS to remove storage fixative. In order to remove excess salts, embryos were washed twice for 10 min in distilled water and subsequently left overnight in distilled water followed by 1 wash of 10 min in fresh distilled water. OPT scanning was carried out following the protocol set out in Sharpe et al. (2002), for more detailed protocols see supplementary materials. The resulting output was in the Wlz file format which the Edinburgh Mouse atlas Project (EMAP) and EMAGE projects have utilised and which can store 3D grey scale information. The MRC HGU has produced a set of software tools for manipulating data in this format and these tools were used to convert the data into a format that could be imported in to the AMIRA software package.

### 3D mapping

The mapping of the 3D gene expression data to the reference models was performed using the AMIRA 4.1 software from Mercury Computer Systems. The data to be mapped were first roughly aligned with the reference model. Two corresponding sets of landmarks were then set up between an isosurface for the reference embryo and an isosurface for the fluorescent/anatomical data from the scan to be mapped. The landmarks were based on prominent morphological landmarks such as the AER, the region where the limb attaches to the flank and to proportional distances along the main axes of the limb. The fluorescent/anatomical data was warped, using a Bookstein thin plate spline method (Bookstein, 1989) provided by the AMIRA software and based on the previously defined landmark sets. Provided the resulting warped fluorescent/anatomical data seemed consistent with the reference limb’s morphology the same warp was then applied to the brightfield channel data. For full details see methods in supplementary materials.

### Real-time PCR

Chick embryos (incubated for 4 days at 38 °C) *i.e.*, approx. stage 22–23, were harvested in ice-cold PBS, the limb buds removed and the distal third cut off with tungsten needles. These pieces were immediately transferred to RNALater (Qiagen). A similar procedure was carried out on the proximal and median thirds. RNA preps were made of pooled limb sections with 20 sections for each region using the Qiagen RNA Easy micro kit, and checked using an Agilent bioanalyzer, using their RNA 6000 nanochip. The integrity values of all RNA samples were between 9.9 and 10 and the 28S and 18S ratio between 1.9 and 2.1. These values indicate little degradation or contamination.

Real-time PCR was carried out using an Applied BioSystems HT-7900 machine in a manner similar to Jesmin et al. (2007) using the FastStart Taqman Probe master (Rox) standard reaction mix (Roche). Primers were selected from the Roche Universal Library using their online software. For the *Wnt5a* reaction, probe 52 was used, and for *β-actin*, probe 43 was used. Here chick probes are not automatically checked against other possible hybridisation targets, so this was carried out manually by Blasting the candidate sequences against the chick genome in Ensembl. The primers used for the *Wnt5a* reaction were: forward 5′catgatgaacctacacaatga 3′; reverse 5′ ccacgtcagccaggttgta 3′. And for the *β-actin* reaction were: F 5′ cacacaagtgcccatttacga 3′; R 5′ caagtccagacgcaggatg 3′. For full protocol see supplementary materials.

### Computational analysis

Simple arithmetical analyses were produced using either the AMIRA software package or Wlz based software tools. AMIRA’s arithmetic module was used to produce averages of multiple datasets and to produce masked datasets for domains of coexpression. Wlz based software developed by the MRC HGU was used to derive medians from multiple datasets and also to derive mean grey level intensity values for both discrete domains and serial sections through the limb. For fuller details of these image manipulations see supplementary materials and methods.

For more complex computational analyses each of the experimental gene-expression spatial distributions has been mapped into the standard coordinate framework defined by the model limb. To analyse the gene-expression patterns we first divided the limb into 560 non-overlapping sub-regions each of 10 × 10 × 10 voxels. Each of these was used to sample the experimental gene-expression patterns. For each experimental pattern, the mean gene-expression strength (integrated optical density divided by the volume) within each box was calculated. If the box was partially external to the limb then only the intersecting volume was considered. By this means a 2D matrix of mean expression strengths across the limb was calculated. Each row of the matrix for a given gene is a low-resolution representation of the pattern and each column for a given sample-region is the genetic “signature” for that spatial location.

The resulting matrix of gene expression values was analysed using the TMEV4 package from TIGR. The data were analysed using a hierarchical clustering method (Eisen et al., 1998) to produce a nested tree of gene expression pattern similarity based on a Euclidean distance metric. A nested tree was also produced of the similarity of the individual sample regions of the limb making up the 3D data model based on gene expression. The resulting tree was then used to identify clusters of similar expression made of small groups of regions at the terminus of long branches. These regions were used to produce larger 3D domains corresponding to the whole volume occupied by the regions comprising each cluster, which were subsequently visualised using the AMIRA software package.

## Results and discussion

### Assessment of efficiency of whole mount *in-situ* hybridisation and scanning with OPT as a method of detecting gene expression domains

An initial technical issue we encountered with OPT scanning of WISH (whole mount *in-situ* hybridisation) specimens was that strong *in-situ* colour reaction staining can block autofluorescence and prevent the capture of portions of the anatomical data required for subsequent mapping to a reference model. To obviate this problem, we identified a particular depth of staining with the NBT–BCIP substrate, suitable for OPT scanning, which captures an extensive range of the expression pattern and allows the visualisation of dynamic gradients without causing a substantial dropout of the anatomical data necessary for mapping (see supplementary materials Fig. S1). To test our standard *in-situ* hybridisation protocol, monitor probe penetration of the embryonic limb, and assess the ability of the OPT system to identify graded patterns of expression within deep tissues, we focussed on *Wnt5a*. *Wnt5a* has been reported to have a proximo-distal gradient of expression based on both radioactive section *in-situ* hybridisation and northern blots of distinct portions of the limb (Dealy et al., 1993) and is known to be expressed throughout mesodermal regions of the limb.

**Fig. 1:**
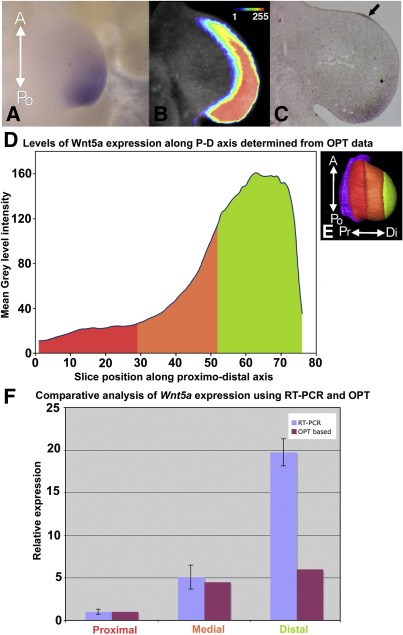
Comparison of WISH/OPT and other methods for detecting a gradient of expression. (A) Dorsal view of wholemount *in-situ* hybridisation of *Wnt5a* in a HH22 wing bud. (B) Virtual section of OPT data scanned from A mapped to a reference limb. (C) Section *in-situ* of Wnt5a from a HH22 wing bud, arrow indicates expression in the AER. (D) A plot of the mean signal intensity in virtual slices of the OPT data set taken along the proximo-distal axis of the limb with 0 representing the most proximal position and 75 the most distal. Colouring under the line represents the domain to which the slices belong, coloured as in panel E. (E) A surface rendering showing the early HH22 reference limb and the three assayed limb domains; proximal (red), medial (orange) and distal (green), arrows indicate the antero-posterior (A-Po) and proximo-distal (Pr-Di) axes of the limb. (F) Comparison of levels of expression in three domains assayed by OPT (purple) and real time RT-PCR (blue), error bars represent standard errors of ± 0.29, ± 1.4 and ± 1.6 for the RT-PCR measurements in the proximal medial and distal regions respectively. OPT values were based on the mean grey level intensity within the domain and standardised against the mean intensity value of the proximal domain to get a relative expression. Domain labels are coloured as in panel E.

**Fig. 2:**
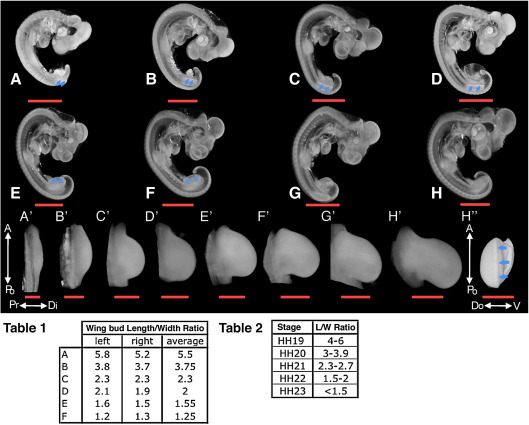
3D reference models of whole embryos over several stages. (A–H) Volume renderings of OPT scans of whole embryos at reference stages HH19 (A), HH20 (B), HH21 (C), Early stage HH22 (D), Late Stage HH22 (E), HH23 (F), HH24 (G), HH25 (H), scale bars (orange) for panels A–H indicate 1 mm. Blue arrows indicate where the AER is visible on rendered embryos. (A′–H′) Plan views of digitally extracted right wing buds from corresponding whole embryo scans. Panel A′ shows arrows indicating the antero-posterior (A-Po) and proximo-distal (Pr-Di) axes of the limb. (H″) Distal view showing AER (blue arrow), white arrows indicate the antero-posterior (A-Po) and dorso-ventral (Do-V) axes of the limb. Scale bars (orange) indicate 300 μm in panels A′–H′ and H″. (Table 1) Measurements of wing bud length to width ratios (*L*/*W*) made on the plan views. (Table 2) Stages according to the original *L*/*W* measurements of Hamburger and Hamilton (1951). The measurements for later stages are not included in Table 1 as they are not covered by Hamburger and Hamilton.

We first assayed expression by WISH (method modified after Nieto et al., 1996 see supplementary data) Fig. 1A). The *Wnt5a* whole mount was scanned using OPT and gene expression data mapped onto a reference limb (Fig. 2D), from which virtual sections were derived (Fig. 1B). These virtual sections were then compared with section *in-situs* (Fig. 1C) performed as in Moorman et al. (2001). This comparison shows that the virtual section captures the extent and range of the *Wnt5a* expression pattern as accurately as the section *in-situ* with the exception of some apical ectodermal ridge (AER) expression (Fig. 1C arrowed). For a further illustration of the effective capture of expression patterns using OPT see supplementary data (Fig. S3–5).

We then measured the mean grey level signal intensity in all individual sections along the proximo-distal axis. The plot of these data (Fig. 1D) shows low levels of signal in the proximal region (Red, slice 1–28), either very low expression or background. The mean grey level intensity then climbs steeply in the medial region (Orange, slice 29–52) from ∼ 30 up to 100. In the distal region (Green, 53–75) the mean grey level intensity increases less steeply and then levels off at a mean intensity of around 160. In the final 5 slices the mean grey level intensity drops rapidly. This shows the capability of the OPT imaging method to allow a detailed analysis of graded patterns of expression.

We also compared *Wnt5a* expression levels as measured from OPT scans of whole mount *in-situs* with real-time RT-PCR analysis (Fig. 1F). For both OPT and RT-PCR analyses the limb bud was divided into three regions of equal length along the proximo-distal axis designated proximal, medial and distal (Fig. 1E). For RT-PCR the sample tissue was dissected into the three regions of equal length and samples from 10 embryos were pooled. In the case of the OPT data this division was performed digitally using AMIRA’s segmentation software based upon guidance from the researcher who performed the initial microdissection. The real time RT-PCR data produced relative values for the expression of *Wnt5a* as follows; expression in the proximal region was taken as the reference expression level, the medial region had a 5 fold increase over the proximal region and the distal region a 19.7 fold increase. The OPT based analysis produced relative values for medial and distal regions of 4.5 fold and 6 fold increases over the value for the proximal region respectively. Therefore, WISH/OPT captures the graded nature of the expression along the proximo-distal axis, indeed the correspondence in the proximal and medial regions is striking, but not across the whole quantitative range captured using RT-PCR as the correspondence falls off dramatically at the higher levels seen in the distal region. The limitations in the captured range of expression may be due to limitations of the WISH detection method, *i.e.*, a nonlinear relationship between signal intensity and RNA quantity. This allows comparisons of the level of expression of a particular gene within a particular scan but not the comparison of absolute levels between different scans, although high and low regions of expression could be compared.

The quality of data capture for the gradient of *Wnt5A*, both from selected domains and from serial virtual sections, suggests that WISH/OPT is suitable for examining complex patterns of graded expression in tissues within developing embryos, but not for quantification of signal with high accuracy and quantitative comparisons of mRNA levels between samples.

### Reference models for comparative analysis

An initial requirement for meaningful comparison of gene expression patterns is a common spatial reference framework onto which different patterns can be mapped. We have produced a panel of such reference frameworks for several embryonic stages; whole embryos, isolated and fixed according to a standardised protocol, were collected at Hamburger Hamilton (HH) (Hamburger and Hamilton, 1951) stages from HH18 through to HH25. Embryos were then scanned by OPT using autofluorescence stimulated by a UV lamp and reconstructed to produce 3D models. These models were rendered using the AMIRA software package to show morphology and gross anatomy of the embryos (Figs. 2A–H). This resulted in a clear 3D visualisation of the embryo and revealed details such as the AER – the thickened layer of epithelium that rims the distal limb buds – in the models from HH20 onwards (Figs. 2A–F, blue arrows indicate AER). This is best appreciated when the model is rotated (Fig. 2H″, blue arrows indicate AER) (to view models in 3D see movies in supplementary data Fig. S6). Measurements of the length (*L*; along a line from anterior join of bud and trunk to posterior join) and width (*W*; a line from distal tip to trunk perpendicular to line *L*) of wing buds were made on the dorsal plan view (Fig. 2, Table 1) and the *L*/*W* ratio calculated in order to stage the reference embryo (Fig. S2). The procedure was the same as one would use to stage a living chick embryo using the staging criteria of Hamburger and Hamilton (Fig. 2, Table 2) (supplementary Fig. S2).

Reference models for specific regions, such as the developing limb buds (Figs. 2A′–H′), can be extracted from whole embryo models and used for mapping of gene expression in these regions. Within the AMIRA program, such extracted models remain in register with the model of the whole embryo from which they are derived, thus allowing maintenance of a consistent positional system between all expression patterns mapped. These relative positions are also maintained when files are exported to the Wlz file format, which can be used to store 3 dimensional greyscale image data, using software tools developed by the MRC HGU. Subsequent mappings and analyses of gene expression reported here were performed on an extracted data set for the right wing bud of the late stage HH22 reference embryo (Fig. 2E′). Further reference sets for other stages can be easily generated.

### Reproducibility of 3D mapping of gene expression within and between labs

To test the reliability of our *in-situ* protocol and 3D mapping, we focussed on the expression domain of *Sonic hedgehog* (*Shh*) in HH22 wing buds. *Shh* is expressed in the polarizing region at the posterior margin of the wing bud and *Shh* expression correlates with maps of polarizing activity (Riddle et al., 1993). We compared data generated from wing buds in a single round of whole mount *in-situ* hybridisation experiments and from wing buds processed in three different labs.

**Fig. 3:**
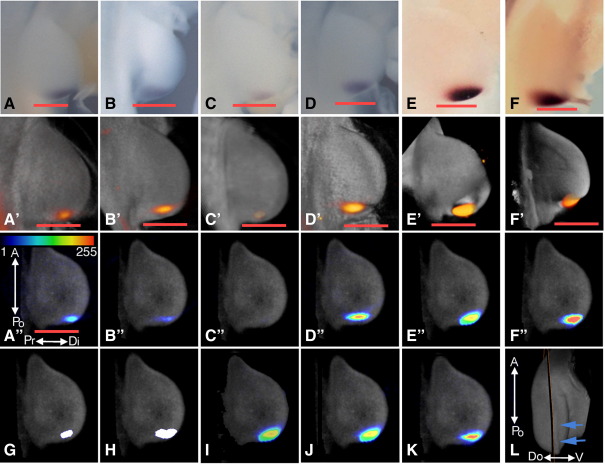
Reproducibility of 3D mapping of gene data. Photographs of right wing buds from four whole mount *in-situ* hybridisations for *Shh* from Dundee (A–D), one from Edinburgh (E) and one from Dublin (F). (A′–F′) OPT scans of the *in-situs* shown in panels A–F using both the fluorescence channel, for the anatomy (grey), and brightfield, for the signal (orange), visualised as volume renderings. (A″–F″) Gene expression data derived from the OPT scans in panels A′–F′ are displayed on a correspondingly labelled view of an A-Po/Pr-Di section (L) of the early HH22 wing bud (Fig. 2D), signal intensity is represented according to the heatmap in panel A″. (G) Intersect of domains of expression, in white, of panels A″–F″. (H) Intersect of domains of expression, in white, of panels A″, B″ and D″–F″, excluding the restrictive outlying data set C. (I) Mean of data in panels A″–F″. (J) Mean of data in panels A″–F″ after normalisation. (K) Median of data in panels A″–F″. The orange scale bar in all panels represents 300 μm, the scale shown in panel A″ is consistent for subsequent panels through to K. (L) A 3D model of the early HH22 wing bud showing the A-Po/Pr-Di plane of section, the nearby AER is indicated by blue arrows.

A round of *in-situ* hybridisations using standardised protocols for *Shh* expression were carried out on 4 embryos and the sense control probe was used on 2 embryos. All 4 embryos assayed using the Shh anti-sense probe were treated identically and detection was carried out in the same tube. Nevertheless there were differences in intensity of staining of *Shh* transcripts in the polarizing region (Figs. 3A–D) with wing buds of one embryo (Fig. 3C) showing very faint staining. Wing buds of control embryos (data not shown) had no visible background staining. *In-situs* of embryos from Edinburgh (Fig. 3E) and Dublin (Fig. 3F) showed similar localisation of *Shh* transcripts in the wing bud and one embryo from each site was then scanned together with the four embryos from the run carried out in Dundee.

All six embryos were OPT scanned through different channels to capture a) autofluorescence to represent anatomy and b) the staining pattern under visible light. Having reconstructed 3D representations of each, we then digitally extracted the right wing bud and accompanying flank using the same spatial parameters. Co-visualisation of both anatomy (grey) and expression (orange) with volume rendering (Figs. 3A′–F′) shows the representation of the original *in-situ* data (Figs. 3A–F) following OPT scanning and reconstruction.

The patterns of *Shh* expression in each of the six wing buds were then mapped in 3D to the HH22 reference wing (Fig. 2E). Figs. 3A″–F″ shows heat maps of intensity of *Shh* expression in one section, taken across the antero-posterior/dorso-ventral (A-Po/Do-V) axes of the HH22 stage reference wing bud in a plane situated next to the AER (Fig. 3L), for the individual patterns of expression for the six source wing buds. Signal intensity of mapped gene expression data is represented by the heatmap in Fig. 3A″ corresponding to grey scale values between 1 and 255, this measure of intensity is not suitable for precise quantitative comparisons between samples but allows visualisation of the differing levels of expression within a sample within the limits of the WISH methodology. Of the 4 wing buds from the Dundee laboratory one showed a weak signal (Fig. 3C″), with a maximal intensity of only 28. The sense controls were both very clean with no signal (data not shown). Scans for Edinburgh (Fig. 3E″) and Dublin (Fig. 3F″) had a localisation very similar to Fig. 3A″ but an intensity of expression much closer to Fig. 3D″.

Both unique domains of expression and intersecting domains of expression can be derived for these data sets. Visualisation of the unique expression domains in 3 dimensions shows no unique domains for the specimens shown in A–D and only small peripheral regions for the higher intensity signal data sets from specimens in E and F (Data not shown). The intersect of the expression domains between all the scans (Fig. 3G) is restricted by the small domain of the outlying data set (Fig. 3C″), if we remove this outlying data from the calculation we have an intersect domain almost twice as large (Fig. 3H).

Clearly, there is some variation between individual scans and an occasional extreme outlier, but our data suggest a clear common domain of gene expression is identifiable. To produce a reliable and robust domain which could compensate for the variations we see in individual *in-situs*, we incorporated the data from our multiple scans into one domain. We produced means of the data from the scans and corrected for background from the controls, using both raw and normalised data (Figs. 3I–J). The mean of the datasets (Fig. 3I) was heavily influenced by high intensity samples (compare Fig. 3E″ and Fig. 3I). To correct for the variation in signal intensity, whether as a result of differences in the *in-situ* itself or from the scanning steps, data sets were normalised by stretching their entire grey value range to cover the maximal range of 0–255. Such correction had almost no effect (Fig. 3J), although there was a small extension of the domain proximally. As an alternative to normalisation to account for variability and extreme outlying values we derived a median value for each voxel based on the grey levels of all of the scans (Fig. 3K). This median value seemed less dominated by outliers and a better representation of the range seen across the differing samples although it produced a more conservative domain than the simple mean since it removed areas where signal was not apparent in more than half of the samples.

These results suggest that best practice for producing a reliable domain of expression for comparison is to perform several *in-situs* developed to suitable stain intensity and merge the resultant data. A minimum of four scans seems advisable to contribute to the merged data set and these may then be mapped to a common reference and a mean or median expression pattern derived. A mean of the patterns appears to better emphasise the extent of the expression domain while the median produces a more conservative domain less affected by outliers. As our analysis shows, more complicated treatment of the data seems to make little difference to the resulting domains although, for *in-situs* with persistently low signal, normalisation might help emphasise gradients of expression in some cases.

This averaging or otherwise merging together of multiple samples is less necessary in the case of previously well characterised genes where the expected pattern of expression is already known and a representative sample can be confidently identified. This approach should be most valuable in the situation where the gene expression is either poorly characterised or unknown, as is likely to be the case in large scale screens.

### Comparative analysis of 3D Gene expression patterns

**Fig. 4:**
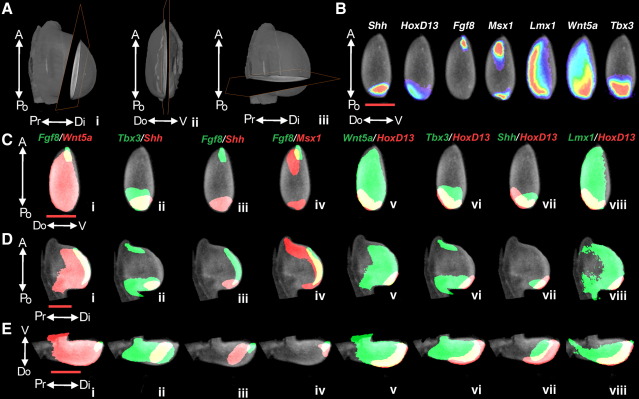
Mapping of gene expression for *Shh*, *HoxD13*, *Fgf8*, *Msx1*, *Lmx*, *Wnt5a* and *Tbx3*. (A) 3 views of the late stage HH22 reference model showing the position of planes of section used for subsequent analyses. (A.i) A dorsal view of the limb showing the position of the A-Po/Do-V plane seen in panels B and C. (A.ii) A distal view of the limb showing the position of the A-Po/Pr-Di plane seen in panel D. (A.iii) A dorsal view of the limb showing the position of the Do-V/Pr-Di plane seen in panel E. (B) A virtual section through the reference limb along the A-Po/Do-V plane shown in panel A.i with mapped gene expression patterns for *Shh*, *HoxD13*, *Fgf8*, *Msx1*, *Lmx1*, *Wnt5a* and *Tbx3*. Sections from the three planes shown in panel A are (C) an A-Po/Do-V plane, (D) an A-Po/Pr-Di plane, (E) a Do-V/Pr-Di plane. Pairwise comparisons were visualised on these planes for expression domains of i) *Fgf8* (green) and *Wnt5a* (red)*,* ii) *Shh* (red) and *Tbx3* (green), iii) *Fgf8* (green) and *Shh* (red), iv) *Fgf8* (green) and *Msx1* (red), v) *Wnt5a* (green) and *HoxD13* (red), vi) *HoxD13* (red) and *Tbx3* (green), vii) *HoxD13* (red) and *Shh* (green), viii) *HoxD13* (red) and *Lmx1* (green). Comparison numberings are consistent between panels C and E*.* Regions of overlap are in yellow. All scale bars (orange) represent 300 μm.

OPT is a rapid method of capturing the 3D expression pattern and can allow data on multiple genes to be integrated into a common framework, therefore we used 3D warping of OPT data to our reference models to produce such an integrated data set. Expression patterns for the genes *Shh*, *HoxD13*, *Fgf8*, *Msx1*, *Lmx1*, *Wnt5a* and *Tbx3* (for representative *in-situs* see supplementary Fig. S6) were mapped to our HH22 reference model (Fig. 2E) using AMIRA’s 3D warping capabilities. This stage was chosen as it represents a well-developed limb bud but still consisting mainly of undifferentiated mesenchyme cells. These genes were chosen for particular characteristics of expression such as dorsal restriction, *Lmx1*, specific expression in the AER, *Fgf8*, specific expression in the mesoderm, *Shh*, or particular gradients of expression, such as the proximo-distal gradient of *Wnt5a*. Particular specimens for scanning were chosen based on *in-situ* quality in comparison to others processed with them, usually the best example from 5–6 *in-situs*. The resulting mappings were then visualised in 3D (see supplementary materials Fig. S8), virtual sections were derived along specific planes (Fig. 4A), gene expression patterns and intensity were visualised on the anterior–posterior/dorsal–ventral plane (Fig. 4A.i). The expression domains of several pairs of genes were co-visualised on the antero-posterior/dorso-ventral (A-Po/Do-V) (Fig. 4C, section plane in Fig. 4A.i), antero-posterior/proximo-distal (A-Po/Pr-Di) (Fig. 4D, section plane in Fig. 4A.ii) and dorso-ventral/proximo-distal (Do-V/Pr-Di) (Fig. 4E, section plane in Fig. 4A.iii) planes to allow some specific comparisons to be drawn (Figs. 4C–E; for 3D visualisations see supplementary materials Fig. S11–18). These mappings show both expected features, such as the dorsal expression pattern of *Lmx1*, and novel features such as an apparent gradient of expression from ventral to dorsal in *Wnt5a*. Indeed several dorso-ventral asymmetries were by far the most striking features to emerge.

One such previously unappreciated asymmetry was in *Shh* expression (Fig. 4B), which shows that *Shh* expression extends further anteriorly on the ventral side of the limb. A transverse section through the limb along plane E shows that the asymmetry is more complex and the domain of *Shh* expression is skewed with the more proximal regions of the limb showing a more dorsal expression of *Shh* (Fig. 4E.ii). Planes taken at more anterior levels lose this obvious skewing (see supplementary Fig. S9) and elements of this distribution are confirmed by both normal *Shh* whole mount *in-situ* and a double *in-situ* for *Shh* and *Fgf8* (see supplementary materials Fig. S10). This may explain the observations of Yang and Niswander (1995) who reported no apparent dorso-ventral asymmetry in *Shh* expression at HH24 based on sectioned whole mount *in-situ* hybridisation data. Alternatively this may be due to the different stages assayed. A more dorsal distribution in the more proximal regions of the limb may suggest that a dorsally localised signal, such as *Wnt7a*, might be maintaining dorsal expression proximally while more distal expression would be maintained evenly across the dorso-ventral axis by signals from the apical ectodermal ridge. Indeed it is already known that Wnt7a plays a role in maintaining *Shh* expression in the polarising region (Parr and McMahon, 1995; Yang and Niswander, 1995). Furthermore Kawakami et al. (1999) reported that *Frizzled10* (a Wnt receptor) colocalises with *Shh* dorsally and suggested that *Shh* expression in this part of the polarising region might be regulated by Wnt7a (Kawakami et al., 2000).

The pairwise comparisons similarly produced both expected and unexpected results. *HoxD13* shows striking asymmetrical dorso-ventral expression (Figs. 4C.v and E.v) and the ventral margin of *HoxD13* expression appears to coincide with that of *Lmx1* (Figs. 4C.viii and E.viii; Movie in supplementary Fig. S18), suggesting a possible regulatory relationship. The dorso-ventral asymmetry in *Hoxd13* had been previously noted by Duboule et al. from 3D reconstructions based on radioactive section *in-situs* (Olivo et al., 1993) but at this time *Lmx1* was not known. Recent lineage tracing studies in the developing mouse limb have shown the existence of a dorsoventral lineage restriction compartment further suggesting that there is still considerable complexity in the dorsoventral organisation of the limb to be discovered (Arques et al., 2007).

Comparison of *Shh* and *Fgf8* expression (Figs. 4D.iii and E.iii; movie in supplementary Fig. S13) shows an unexpected overlap of expression in the mesoderm. This shows that there are important limits of spatial resolution to the current mapping procedure given the well-characterised expression of these genes in mesoderm and apical ectodermal ridge respectively. Since the AER is one of the principle morphological features of the limb, it is heavily used in the landmarking process, which is the first stage of mapping expression data to the reference limb. Strong *in-situ* staining, as seen with *Fgf8*, can block autofluorescence and prevent the capture of the anatomical data for the ridge. A comparison of *Wnt5a* and *Fgf8* (Movie in supplementary Fig. S11) also shows considerable overlap except for the most anterior region of *Fgf8* expression (Fig. 4C.i) and a persistent ‘leading edge’ of *Fgf8* expression in the most distal portion of the limb (Fig. 4E.i). In this case, we would expect to see considerable overlap due to expression of *Wnt5a* in the ridge, although it is not clear whether the common domain of expression accurately represents just ridge expression.

### OPT using Tyramide Signal Amplification

**Fig. 5:**
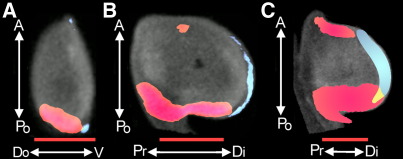
Improved localisation of *Fgf8* using fluorescent *in-situ* hybridisation. (A) An A-Po/Do-V plane through the HH24 reference limb (Fig. 2G′), (B) An A-Po/Pr-Di plane through the HH24 reference limb and (C) An A-Po/Pr-Di plane through the HH22 reference limb as in Fig. 4D. All 3 panels show expression of both *Fgf8* (red) and *Tbx3* (blue) and the overlap of the domains of expression (yellow). Expression data for *Tbx3* are NBT–BCIP derived in all cases. *Fgf8* expression data in panels A and B are derived from Tyramide signal amplified FISH and NBT–BCIP derived in panel C. Note marked reduction in overlap in panels A and B compared with panel C.

To address the problem of the loss of anatomical landmarks when looking at strongly expressed ectodermal genes, such as *Fgf8*, we used a Tyramide Signal Amplification (TSA) kit (Invitrogen) to enhance a fluorescent colour reaction and avoid the blocking effect seen with chromagenic substrates such as NBT–BCIP. We compared the expression of *Tbx3* and *Fgf8* at HH24, mapped to the HH24 reference limb (Fig. 2G′), from a normal NBT–BCIP based colour reaction for *Tbx3* and a TSA fluorescent colour reaction for *Fgf8* (Figs. 5A, B). This approach provided a much more accurate localisation of *Fgf8* expression to the apical ridge compared to that seen in Fig. 4. When we carried out a pairwise comparison between *Fgf8* and *Tbx3* expression patterns, the overlap was very much reduced compared to that seen between *Fgf8* and *Shh* in Fig. 4D.iii and compared with *Fgf8* and *Tbx3* from our original data set in which we had used an NBT–BCIP reaction for both genes (Fig. 5C). While these mapped localisations are not sufficient for accurately discriminating expression from expressing tissues close together or very thin tissue layers a database containing such mapped data would be linked to the original 3D scans allowing visualisation of the data.

### Computational comparisons of mapped gene expression patterns

Despite the shortcomings discussed above, our overall mapping strategy provides a valuable tool for analysing spatial and temporal relationships of multiple complex patterns.

To begin to apply computational methods to analyse these multiple data sets at once and to look for similarities in patterns of gene expression, we utilised software produced by the MRC HGU to manipulate 3D image data in the Wlz format, a format used by EMAGE. AMIRA files were converted to Wlz format and a set of sub-regions of 10 × 10 × 10 voxels were defined for the early HH22 reference limb producing a coarse sampling of the 3D model. The expression data for the previously analysed genes and an additional gene, *Wnt3a*, were then used to derive mean expression values from the grey levels of each OPT scan for the newly defined volumes. This produced a matrix of common positional IDs and expression levels for each gene.

**Fig. 6:**
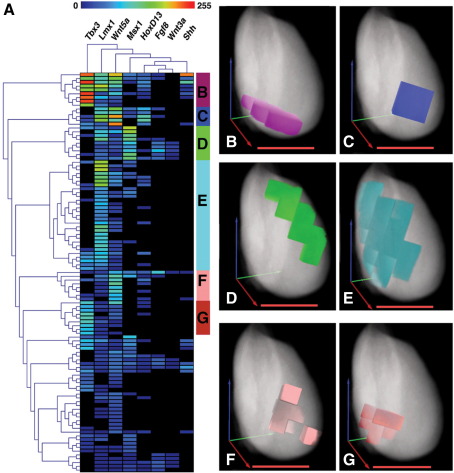
Computational analysis of gene expression data. (A) A hierarchical clustering of the coarse sampled gene expression pattern data for both genes and limb regions. Each column represents a gene expression pattern and each cell within a column of the matrix grid represents a discrete 10 × 10 × 10 spatial volume in the reference model. Both the gene and the spatial volume data have been hierarchically clustered and trees derived showing similarity relationships for genes at the top and for spatial volumes to the left. Cells in the matrix grid are coloured according to the accompanying heatmap for signal intensity and represent the mean signal intensity for a particular gene within a particular spatial volume. This matrix does not show data for all volumes in the reference model. To the right of the matrix are blocks of colour corresponding to clusters located at the end of particularly long branches of the hierarchical clustering tree to the left, the lettered clusters correspond to the visualisations in the subsequent panels. (B–G) Visualisations of the lettered clusters from panel A in a volume rendered view of the limb, anatomy is rendered in grey and the clusters are rendered in the colours corresponding to their labelling on the right hand side of the matrix (B) purple, (C) blue, (D) green, (E) Cyan,(F) pink, (G) red. (A movie showing these clusters is available in the supplemental materials Fig. S19). Scale bars in orange correspond to 300 μm; arrows to orient the 3 principal axes, A-Po axis (blue), Pr-Di axis (red) and Do-V axis (green). Intersect of arrows represents posterior, proximal and dorsal ends of the axes.

These data were then analysed using hierarchical clustering for both the genes and the regions defined by the coarse sampling. This analysis was performed and visualised using the TMEV package (http://www.tm4.org/mev.html) (Fig. 6A). The clustering of these genes fits our expectations with *Fgf8* and *Wnt3a*, both known to be expressed in the apical ridge (Barrow et al., 2003; Kengaku et al., 1998; McQueeney et al., 2002), treeing out together. Visualisation of the clustered regions shows that they largely form contiguous spatial domains (Figs. 6B–G; supplementary Fig. S19); some of these domains are associated with specific regions of the limb. Cluster B lies in a plane through the limb along the proximal–distal axis at the level of the anterior margin of *Shh* expression (Fig. 6B), cluster D is associated with the AER in the anterior of the limb (Fig. 6D) while cluster E is in the dorsal margin of the limb (Fig. 6E). None of the clusters visualised here corresponds simply to the expression pattern of one particular gene.

This simple level of clustering computational analysis shows the potential for methods developed to study gene expression data from other sources, such as microarray data, to be applied to the study of 3D gene expression data. Not only can we identify similarly expressed genes using this method but it should also be possible to identify specific regions of the limb that may be important in the regulation of gene expression, such as novel signalling centres.

## Conclusion

We have developed improved technology for the production of 3D atlases of gene expression (Baldock et al., 2003). Specifically, we have shown that OPT is a reliable and efficient way of visualising 3D patterns of gene expression in the chick limb and have been able to directly compare different patterns on reference models using a 3D warping technique. This technique allows visualisation both of samples too large for confocal microscopy (Welten et al., 2006) and those too small for good resolution with microMRI (see Li et al., 2007 for visualisation of chick wings at later stages). Furthermore, visualisation of gene expression is still rudimentary with microMRI (Liu et al., 2007a).

As more 3D patterns of gene expression are mapped, simple pairwise comparisons will no longer be sufficient to analyse more complex relationships between groups of genes and then the computational approaches we have used here will be greatly beneficial, indeed similar methods have been used to study the expression of around 20,000 genes in the mouse brain (Lein et al., 2007; Liu et al., 2007b). Although we have only compared 7 genes known to be expressed in chick limb development we have already revealed some previously unappreciated asymmetries and relationships, particularly with respect to the dorso-ventral axis. These techniques will also be more generally applicable to different developing structures and organisms.

## Appendix A Supplementary data

Fig. S1 Suggested standard depth of colour development for OPT scanning. HH22 *Wnt5a in-situ* hybridisation showing suggested stain development. B) A colourstrip with the suggested endpoint stain depth highlighted in red.

Fig. S2 This graph compares the measurements of wing bud length/width ratios from reference embryos (Figs. 2A′–F′) to the original values stipulated by Hamburger and Hamilton. The reference model measurements are shown in blue and the error bar represents the variation between the left and right wing buds of the reference embryos. The Hamilton–Hamburger measurements are shown in red and the error bars represent the range of values Hamburger and Hamilton associate with specific stages. Embryos with *L*/*W* values outside the range covered by the Hamburger Hamilton series are not shown on this graph.

Fig. S3 Efficiency of data capture using OPT. HH Stage 22 whole mount *in-situ* hybridisation showing *Msx1* gene expression. B) Raw image data of embryo during OPT data capture. C) Processed image data showing combined brightfield (Red) and fluorescence (Green) channels.

Fig. S4 Raw movie of embryo rotating during OPT data capture.

Fig. S5 Movie showing rotating combined data for the brightfield (Red) and fluorescence (Green) channels.

Fig. S6 Movie showing the rotation of the HH25 stage embryo from Fig. 2H, the AER is readily appreciable on all limbs.

Fig. S7 Whole mount *in-situs* of HH22 embryos assayed for the expression of A) *Msx1*, B) *Lmx1*, C) *Wnt5a*, D) *Wnt3a*, E) *Tbx3*, F) *HoxD13*, G) *Shh* and H) *Fgf8*.

Fig. S8 A movie showing an initial surface render of the anatomy of the limb bud which then fades to reveal a surface rendering of the domain of *Shh* (Red) expression is shown, this is then joined by the domains of *HoxD13* (Purple), *Fgf8* (Green), *Tbx3* (Yellow), *Msx1* (Pink), *Wnt5a* (Cyan) and *Lmx1* (Blue) in sequence.

Fig. S9 A movie showing serial virtual sections along the A-Po/Do-V plane going from distal to proximal through the HH22 reference limb. The expression pattern of *Shh* is displayed on these sections according to the heatmap in Fig. 3A″. The dorsal bias of *Shh* in more proximal sections is apparent.

Fig. S10 Asymmetry of *Shh* expression. A) A distal view of a HH stage 23 *in-situ* showing the expression of *Shh*. The expression is predominantly in the dorsal region of the limb with respect to the AER, indicated by the blue arrow. B) A dorsal view of a HH Stage 23 double *in-situ* for *Fgf8* and *Shh*, both developed with NBT–BCIP. *Shh* expression in the ZPA indicated with red arrow. C) A posterior view of the same HH Stage 23 double *in-situ* for *Fgf8* and *Shh*. The domain of *Shh* expression, indicated by the red arrow, is clearly shifted to the dorsal side of the limb relative to the AER as marked by the expression of *FGF8*.

Fig. S11 A) Movie showing rotation of HH22 reference limb displaying expression domains of both *Wnt5a* (Cyan) and *Fgf8* (Green).

Fig. S12 Movie showing rotation of HH22 reference limb displaying expression domains of both *Tbx3* (Yellow) and *Shh* (Red).

Fig. S13 Movie showing rotation of HH22 reference limb displaying expression domains of both *Shh* (Red) and *Fgf8* (Green).

Fig. S14 Movie showing rotation of HH22 reference limb displaying expression domains of both *Msx1* (Pink) and *Fgf8* (Green).

Fig. S15 Movie showing rotation of HH22 reference limb displaying expression domains of both *Wnt5a* (Cyan) and *HoxD13* (Purple).

Fig. S16 Movie showing rotation of HH22 reference limb displaying expression domains of both *Tbx3* (Yellow) and *HoxD13* (Purple).

Fig. S17 Movie showing rotation of HH22 reference limb displaying expression domains of both *Shh* (Red) and *HoxD13* (Purple).

Fig. S18 Movie showing rotation of HH22 reference limb displaying expression domains of both *Lmx1* (Blue) and *HoxD13* (Purple).

Fig. S19 Movie showing volume renderings of domains representing clusters of gene expression. The clusters appear in alphabetical order relating to their labelling in Figs. 5B–G and are coloured as in Fig. 5.

Paper Materials and methods revised

## References

[bib1] Albertson R.C., Kocher T.D. (2001). Assessing morphological differences in an adaptive trait: a landmark-based morphometric approach. J. Exp. Zool..

[bib2] Arques C.G., Doohan R., Sharpe J., Torres M. (2007). Cell tracing reveals a dorsoventral lineage restriction plane in the mouse limb bud mesenchyme. Development.

[bib3] Baldock R.A., Bard J.B., Burger A., Burton N., Christiansen J., Feng G., Hill B., Houghton D., Kaufman M., Rao J., Sharpe J., Ross A., Stevenson P., Venkataraman S., Waterhouse A., Yang Y., Davidson D.R. (2003). EMAP and EMAGE: a framework for understanding spatially organized data. Neuroinformatics.

[bib4] Barrow J.R., Thomas K.R., Boussadia-Zahui O., Moore R., Kemler R., Capecchi M.R., McMahon A.P. (2003). Ectodermal Wnt3/beta-catenin signaling is required for the establishment and maintenance of the apical ectodermal ridge. Genes Dev..

[bib5] Bell G.W., Yatskievych T.A., Antin P.B. (2004). GEISHA, a whole-mount in situ hybridization gene expression screen in chicken embryos. Dev. Dyn..

[bib6] Boardman P.E., Sanz-Ezquerro J., Overton I.M., Burt D.W., Bosch E., Fong W.T., Tickle C., Brown W.R., Wilson S.A., Hubbard S.J. (2002). A comprehensive collection of chicken cDNAs. Curr. Biol..

[bib7] Bookstein F. (1989). Principal warps: thin-plate splines and the decomposition of deformations. IEEE Trans. Pattern Anal. Mach. Intell..

[bib8] Brown W.R., Hubbard S.J., Tickle C., Wilson S.A. (2003). The chicken as a model for large-scale analysis of vertebrate gene function. Nat. Rev., Genet..

[bib9] Bruner E., Saracino B., Ricci F., Tafuri M., Passarello P., Manzi G. (2004). Midsagittal cranial shape variation in the genus Homo by geometric morphometrics. Coll. Antropol..

[bib10] Carre W., Wang X., Porter T.E., Nys Y., Tang J., Bernberg E., Morgan R., Burnside J., Aggrey S.E., Simon J., Cogburn L.A. (2006). Chicken genomics resource: sequencing and annotation of 35,407 ESTs from single and multiple tissue cDNA libraries and CAP3 assembly of a chicken gene index. Physiol. Genomics.

[bib11] Crossley P.H., Minowada G., MacArthur C.A., Martin G.R. (1996). Roles for FGF8 in the induction, initiation, and maintenance of chick limb development. Cell.

[bib12] Dealy C.N., Roth A., Ferrari D., Brown A.M., Kosher R.A. (1993). Wnt-5a and Wnt-7a are expressed in the developing chick limb bud in a manner suggesting roles in pattern formation along the proximodistal and dorsoventral axes. Mech. Dev..

[bib13] DeLaurier A., Schweitzer R., Logan M. (2006). Pitx1 determines the morphology of muscle, tendon, and bones of the hindlimb. Dev. Biol..

[bib14] Echelard Y., Epstein D.J., St-Jacques B., Shen L., Mohler J., McMahon J.A., McMahon A.P. (1993). Sonic hedgehog, a member of a family of putative signaling molecules, is implicated in the regulation of CNS polarity. Cell.

[bib15] Eisen M.B., Spellman P.T., Brown P.O., Botstein D. (1998). Cluster analysis and display of genome-wide expression patterns. Proc. Natl. Acad. Sci. U. S. A..

[bib16] Hamburger V., Hamilton H.L. (1951). A series of normal stages in the development of the chick embryo. J. Morphol..

[bib17] Harmon E.H. (2007). The shape of the hominoid proximal femur: a geometric morphometric analysis. J. Anat..

[bib18] Hill R.E., Jones P.F., Rees A.R., Sime C.M., Justice M.J., Copeland N.G., Jenkins N.A., Graham E., Davidson D.R. (1989). A new family of mouse homeo box-containing genes: molecular structure, chromosomal location, and developmental expression of Hox-7.1. Genes Dev..

[bib19] Hubbard S.J., Grafham D.V., Beattie K.J., Overton I.M., McLaren S.R., Croning M.D., Boardman P.E., Bonfield J.K., Burnside J., Davies R.M., Farrell E.R., Francis M.D., Griffiths-Jones S., Humphray S.J., Hyland C., Scott C.E., Tang H., Taylor R.G., Tickle C., Brown W.R., Birney E., Rogers J., Wilson S.A. (2005). Transcriptome analysis for the chicken based on 19,626 finished cDNA sequences and 485,337 expressed sequence tags. Genome Res..

[bib20] International Chicken Genome Sequencing Consortium (2004). Sequence and comparative analysis of the chicken genome provide unique perspectives on vertebrate evolution. Nature.

[bib21] Isaac A., Rodriguez-Esteban C., Ryan A., Altabef M., Tsukui T., Patel K., Tickle C., Izpisua-Belmonte J.C. (1998). Tbx genes and limb identity in chick embryo development. Development.

[bib22] Jesmin S., Zaedi S., Shimojo N., Iemitsu M., Masuzawa K., Yamaguchi N., Mowa C.N., Maeda S., Hattori Y., Miyauchi T. (2007). Endothelin antagonism normalizes VEGF signaling and cardiac function in STZ-induced diabetic rat hearts. Am. J. Physiol.: Endocrinol. Metab..

[bib23] Kawakami Y., Wada N., Nishimatsu S.I., Ishikawa T., Noji S., Nohno T. (1999). Involvement of Wnt-5a in chondrogenic pattern formation in the chick limb bud. Dev. Growth Differ..

[bib24] Kawakami Y., Wada N., Nishimatsu S., Nohno T. (2000). Involvement of frizzled-10 in Wnt-7a signaling during chick limb development. Dev. Growth Differ..

[bib25] Kengaku M., Capdevila J., Rodriguez-Esteban C., De La Pena J., Johnson R.L., Belmonte J.C., Tabin C.J. (1998). Distinct WNT pathways regulating AER formation and dorsoventral polarity in the chick limb bud. Science.

[bib26] Kerwin J., Scott M., Sharpe J., Puelles L., Robson S.C., Martinez-de-la-Torre M., Ferran J.L., Feng G., Baldock R., Strachan T., Davidson D., Lindsay S. (2004). 3 dimensional modelling of early human brain development using optical projection tomography. BMC Neurosci..

[bib27] Kim H., Lim D., Han B.K., Sung S., Jeon M., Moon S., Kang Y., Nam J., Han J.Y. (2006). ChickGCE: a novel germ cell EST database for studying the early developmental stage in chickens. Genomics.

[bib28] Lee K., Avondo J., Morrison H., Blot L., Stark M., Sharpe J., Bangham A., Coen E. (2006). Visualizing plant development and gene expression in three dimensions using optical projection tomography. Plant Cell.

[bib29] Lein E.S., Hawrylycz M.J., Ao N., Ayres M., Bensinger A., Bernard A., Boe A.F., Boguski M.S., Brockway K.S., Byrnes E.J., Chen L., Chen L., Chen T.M., Chin M.C., Chong J., Crook B.E., Czaplinska A., Dang C.N., Datta S., Dee N.R., Desaki A.L., Desta T., Diep E., Dolbeare T.A., Donelan M.J., Dong H.W., Dougherty J.G., Duncan B.J., Ebbert A.J., Eichele G., Estin L.K., Faber C., Facer B.A., Fields R., Fischer S.R., Fliss T.P., Frensley C., Gates S.N., Glattfelder K.J., Halverson K.R., Hart M.R., Hohmann J.G., Howell M.P., Jeung D.P., Johnson R.A., Karr P.T., Kawal R., Kidney J.M., Knapik R.H., Kuan C.L., Lake J.H., Laramee A.R., Larsen K.D., Lau C., Lemon T.A., Liang A.J., Liu Y., Luong L.T., Michaels J., Morgan J.J., Morgan R.J., Mortrud M.T., Mosqueda N.F., Ng L.L., Ng R., Orta G.J., Overly C.C., Pak T.H., Parry S.E., Pathak S.D., Pearson O.C., Puchalski R.B., Riley Z.L., Rockett H.R., Rowland S.A., Royall J.J., Ruiz M.J., Sarno N.R., Schaffnit K., Shapovalova N.V., Sivisay T., Slaughterbeck C.R., Smith S.C., Smith K.A., Smith B.I., Sodt A.J., Stewart N.N., Stumpf K.R., Sunkin S.M., Sutram M., Tam A., Teemer C.D., Thaller C., Thompson C.L., Varnam L.R., Visel A., Whitlock R.M., Wohnoutka P.E., Wolkey C.K., Wong V.Y. (2007). Genome-wide atlas of gene expression in the adult mouse brain. Nature.

[bib30] Li X., Liu J., Davey M.G., Duce S., Jaberi J., Liu G., Davidson G., Tenent S., Mahood R., Brown P., Cunningham C., Bain A., Beattie K., McDonald L., Schmidt K., Towers M., Tickle C., Chudek J.A. (2007). Micro-magnetic resonance imaging of avian embryos. J. Anat..

[bib31] Liu C.H., Kim Y.R., Ren J.Q., Eichler F., Rosen B.R., Liu P.K. (2007). Imaging cerebral gene transcripts in live animals. J. Neurosci..

[bib32] Liu Z., Yan S.F., Walker J.R., Zwingman T.A., Jiang T., Li J., Zhou Y. (2007). Study of gene function based on spatial co-expression in a high-resolution mouse brain atlas. BMC Syst. Biol..

[bib33] Maniatis T., Fritsch E., Sambroook J. (1982). Molecular Cloning: A Laboratory Manual.

[bib34] McGurk L., Morrison H., Keegan L.P., Sharpe J., O’Connell M.A. (2007). Three-dimensional imaging of *Drosophila melanogaster*. PLoS ONE.

[bib35] McQueeney K., Soufer R., Dealy C.N. (2002). Beta-catenin-dependent Wnt signaling in apical ectodermal ridge induction and FGF8 expression in normal and limbless mutant chick limbs. Dev. Growth Differ..

[bib36] Moorman A.F., Houweling A.C., de Boer P.A., Christoffels V.M. (2001). Sensitive nonradioactive detection of mRNA in tissue sections: novel application of the whole-mount in situ hybridization protocol. J. Histochem. Cytochem..

[bib37] Nieto M.A., Patel K., Wilkinson D.G. (1996). In situ hybridization analysis of chick embryos in whole mount and tissue sections. Methods Cell Biol..

[bib46] Olivo J., Izpisúa Belmonte J.C., Tickle C., Boulin C., Duboule D. (1993). Reconstruction from serial sections: a tool for developmental biology. Application to Hox genes expression in chicken wing buds. BioImaging.

[bib38] Parr B.A., McMahon A.P. (1995). Dorsalizing signal Wnt-7a required for normal polarity of D-V and A-P axes of mouse limb. Nature.

[bib39] Riddle R.D., Johnson R.L., Laufer E., Tabin C. (1993). Sonic hedgehog mediates the polarizing activity of the ZPA. Cell.

[bib40] Sharpe J., Ahlgren U., Perry P., Hill B., Ross A., Hecksher-Sorensen J., Baldock R., Davidson D. (2002). Optical projection tomography as a tool for 3D microscopy and gene expression studies. Science.

[bib41] Tickle C. (2004). The contribution of chicken embryology to the understanding of vertebrate limb development. Mech. Dev..

[bib42] Verbeek F.J., den Broeder M.J., Boon P.J.B.B., Doerry E., van Raaij E.J., Zivkovic D. (1999). Standard 3D digital atlas of zebrafish embryonic development for projection of experimental data. Proc. SPIE.

[bib43] Visel A., Thaller C., Eichele G. (2004). GenePaint.org: an atlas of gene expression patterns in the mouse embryo. Nucleic Acids Res..

[bib47] Welten M.C., de Haan S.B., van den Boogert N., Noordermeer J.N., Lamers G.E., Spaink H.P., Meijer A.H., Verbeek F.J. (2006). zebraFISH: Fluorescent in situ hybridization protocol and 3D imaging of gene expression patterns. Zebrafish.

[bib44] Yang Y., Niswander L. (1995). Interaction between the signaling molecules WNT7a and SHH during vertebrate limb development: dorsal signals regulate anteropo7sterior patterning. Cell.

[bib45] Yeh J. (2002). The effect of miniaturized body size on skeletal morphology in frogs. Evol. Int. J. Org. Evol..

