# Supplementary figures and images for: Integrating technologies for comparing 3D gene expression domains in the developing chick limb
Source: Dev Biol. 2008 May 1;317(1):13–23. doi: 10.1016/j.ydbio.2008.01.031 (PMC2529376; doi:10.1016/j.ydbio.2008.01.031)

**A**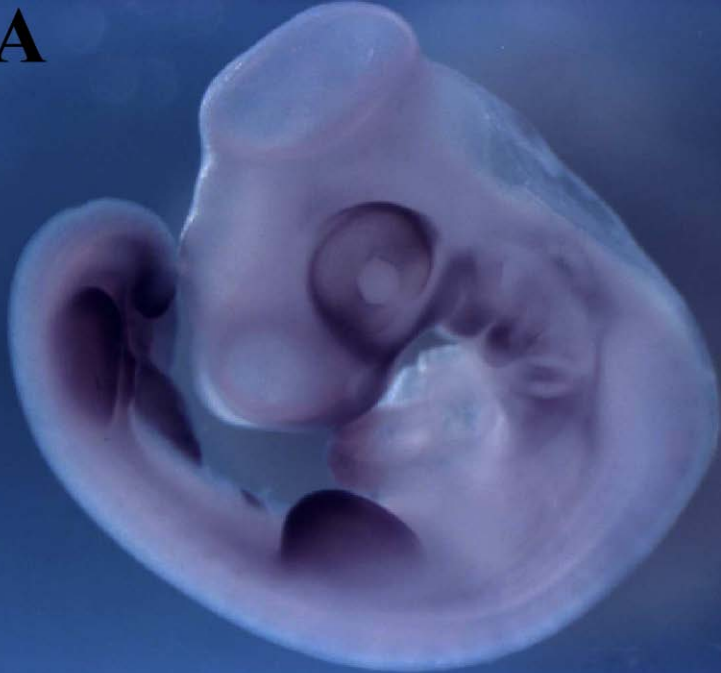**B**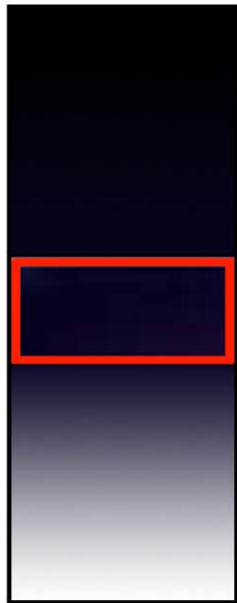

Supplement: Fig. S1 — Suggested standard depth of colour development for OPT scanning. HH22 Wnt5a in-situ hybridisation showing suggested stain development. B) A colourstrip with the suggested endpoint stain depth highlighted in red. [file mmc1.pdf]

Length-width ratio measurements in reference embryos and Hamburger-Hamilton normal stages

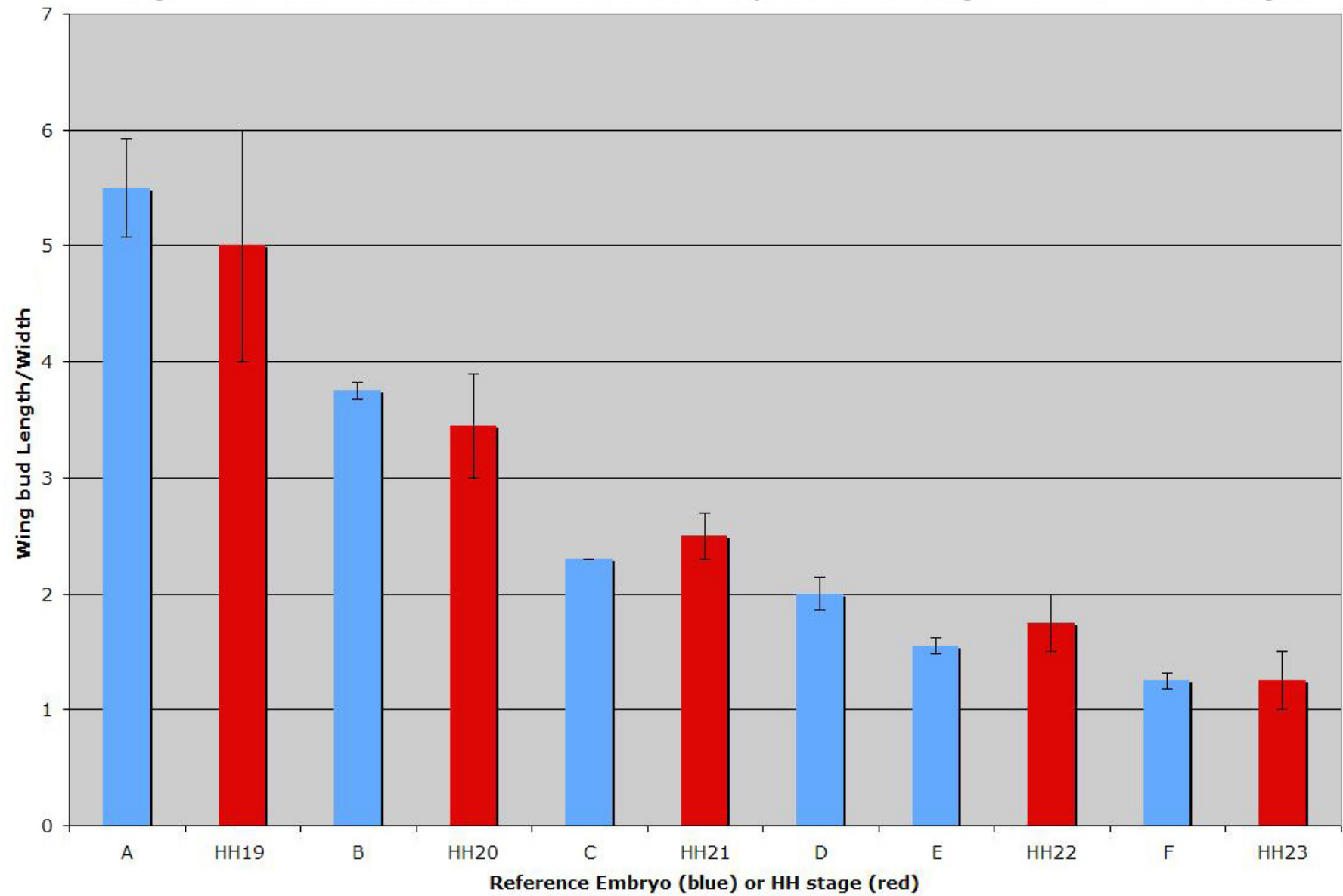

Supplement: Fig. S2 — This graph compares the measurements of wing bud length/width ratios from reference embryos (Figs. 2A′–F′) to the original values stipulated by Hamburger and Hamilton. The reference model measurements are shown in blue and the error bar represents the variation between the left and right wing buds of the reference embryos. The Hamilton–Hamburger measurements are shown in red and the error bars represent the range of values Hamburger and Hamilton associate with specific stages. Embryos with L/W values outside the range covered by the Hamburger Hamilton series are not shown on this graph. [file mmc2.pdf]

**A**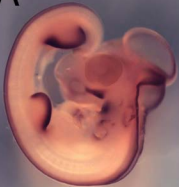**B**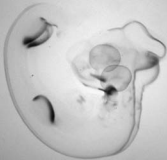**C**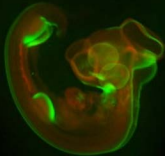

Supplement: Fig. S3 — Efficiency of data capture using OPT. HH Stage 22 whole mount in-situ hybridisation showing Msx1 gene expression. B) Raw image data of embryo during OPT data capture. C) Processed image data showing combined brightfield (Red) and fluorescence (Green) channels. [file mmc3.pdf]

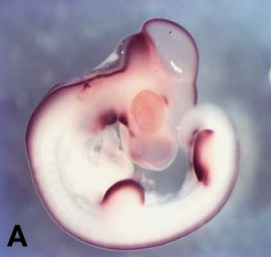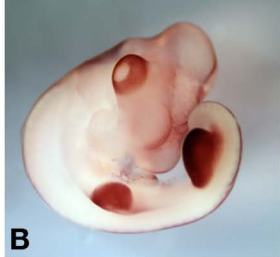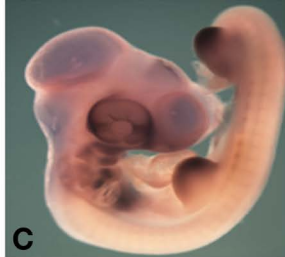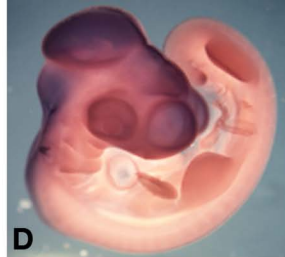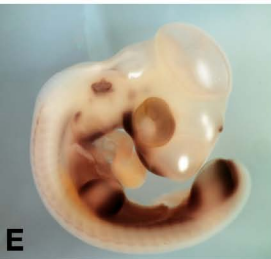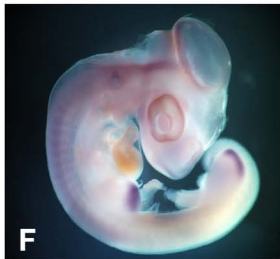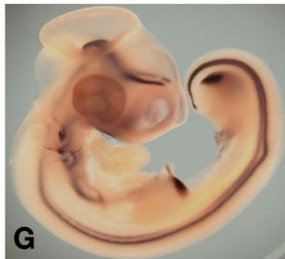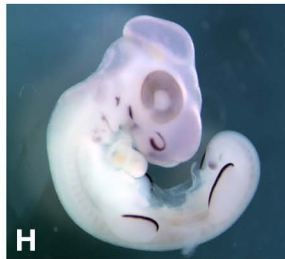

Supplement: Fig. S7 — Whole mount in-situs of HH22 embryos assayed for the expression of A) Msx1, B) Lmx1, C) Wnt5a, D) Wnt3a, E) Tbx3, F) HoxD13, G) Shh and H) Fgf8. [file mmc7.pdf]

**A**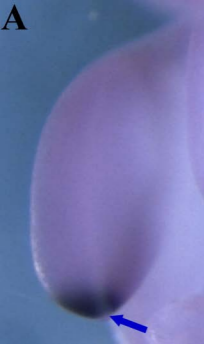**B**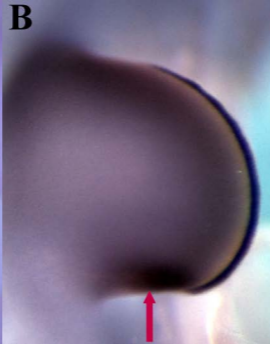**C**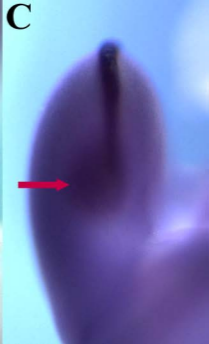

Supplement: Fig. S10 — Asymmetry of Shh expression. A) A distal view of a HH stage 23 in-situ showing the expression of Shh. The expression is predominantly in the dorsal region of the limb with respect to the AER, indicated by the blue arrow. B) A dorsal view of a HH Stage 23 double in-situ for Fgf8 and Shh, both developed with NBT–BCIP. Shh expression in the ZPA indicated with red arrow. C) A posterior view of the same HH Stage 23 double in-situ for Fgf8 and Shh. The domain of Shh expression, indicated by the red arrow, is clearly shifted to the dorsal side of the limb relative to the AER as marked by the expression of FGF8. [file mmc10.pdf]
