# Supplementary material for: Integrating technologies for comparing 3D gene expression domains in the developing chick limb
Source: Dev Biol. 2008 May 1;317(1):13–23. doi: 10.1016/j.ydbio.2008.01.031 (PMC2529376; doi:10.1016/j.ydbio.2008.01.031)
Supplement: Paper Materials and methods revised [file mmc20.doc]

**Supplementary: Materials and Methods**

**Embryo preparation:**

White Leghorn chick eggs were incubated in a humidified incubator at 38^o^C

for the appropriate time for the desired developmental stage. Eggs were then

windowed and embryos removed to ice-cold Phosphate Buffered Saline

(PBS) (0.02 M phosphate, 0.15 M NaCl) and cleaned of extra-embryonic

membranes. Eyes and forebrain were punctured with a tungsten needle to

reduce trapping, and transferred overnight to 4% (w/v) ice-cold

paraformaldehyde (PFA). The embryos were then put through a graded

methanol series at 4^o^C; ending in 2, 100% methanol washes and stored at

-20^o^C until use.

**Probe plasmids and Plasmid Preparation:**

Plasmid clones of the genes to be studied were grown up using standard

protocols. This involved plating out transformed bacteria containing the

plasmids onto L-broth/ampicilin plates and isolation of plasmids from

individual colonies using Qiagen plasmid mini kits.

The plasmids used for the different genes were *Shh* as in Echelard et

al. (Echelard et al., 1993), *Fgf8* as in Crossley et al. (Crossley et al., 1996),

*Msx1* as in Hill et al.(Hill et al., 1989), and*Tbx3* as in Isaac et al.(Isaac et al.,

1998). EST clones acquired from ARK genomics were used as probes for

*Wnt3a* (EST clone 603102629F1), *Lmx1* (EST clone 603127966F1), *Wnt5a*

(EST clone 603799237F1) and *HoxD13* (EST clone 603499362F1). All EST

clones were in pBluescript II KS+, which was linearised with Not1 (NEB) and

transcribed with T3 RNA polymerase (Roche) to produce antisense probes.

Clones were sequenced to check their identity.

**Probe Synthesis:**

Linear plasmid DNA fragments were produced by cutting 10-20 μg of the

plasmid with the appropriate restriction enzyme, and precipitating the DNA by

the addition of acetate/ethanol following standard procedures (Maniatis et al.,

1982). After cooling to –20^o^C for <1 hr. the cleaned DNA fragments were

pelleted in a microfuge for 20 mins., washed in 80%(v/v) ethanol and vacuum

dried. The fragments were then resuspended in ~10 μl of TE (10mM tris-Cl pH

7.0;1mM EDTA) and an aliquot quantified by running out on an agarose gel

(~0.7%w/v) with ~1μg of lambda DNA marker.

RNA probe was prepared in reactions containing T3/T7 RNA polymerase enzyme buffer; 100mM DTT, Dig-NTP mix (Roche), 100U RNase inhibitor (Roche), 1μg linear DNA fragments and 10U of the appropriate RNA

polymerase (Roche). After incubation at 37^o^C for 1 hr. a tenth of the reaction

was run on an agarose gel to quantify the RNA product (with respect to the

linear starting material). If sufficient RNA had been produced, the probe was

purified using the ProbeQuant G-50 spin column system (Amersham

Biosciences). In some cases probe purification was performed using phenol

chloroform extraction and Lithium Chloride precipitation as detailed in Nieto et

al. (Nieto et al., 1996).

**Whole Mount *In-situ* hybridisation:**

Whole mount *In-situ* hybriidsations were performed using a modified form of

the method of Nieto et al. (Nieto et al., 1996). Embryos were rehydrated

through a graded series of 4^o^C MeOH washes into 100% PBT (PBS/0.1%

TWEEN20). Embryos were treated with 20 μg /ml proteinase K in PBT for ~20

mins at room temperature. Embryos were washed a further 3 times in PBT

and re-fixed in 4% PFA/0.2% glutaraldehyde for 20 mins. They were then

rinsed a further 3 times in PBT at room temperature before being put into

prewarmed PBT and incubated at 65^o^C for half an hour. This step was found

to reduce background and increase penetration for a wider range of probes

than studied here. Embryos were transferred to hybridisation buffer at 65^o^C

where they were allowed to equilibrate until they sank. Embryos were then

prehybridised in fresh hybridisation buffer at 65^o^C for at least 1hr. After

prehybridisation embryos were transferred to fresh hybridisation buffer and ~

1ng of probe was added per μl of hyb. mix. Following overnight hybridisation

at 65^o^C, the hybridisation buffer containing the probe was removed for reuse

and embryos were washed for 10 minutes in fresh hybridisation buffer at

65oC to remove unbound probe prior to the SSC washes. SSC washes are

as follows; 2 washes of 10 minutes in 2xSSC, followed by 3 washes of 20

minutes in 2xSSC/0.1% CHAPS and 3 washes of 20 minutes in

0.2xSSC/0.1% CHAPS, all at 65^o^C. Embryos were then washed twice in

MABT (Maleic acid buffer/ 0.1% TWEEN20) at room temperature for 10

minutes. Embryos were then immersed in blocking buffer (3% Boehringer

Blocking Reagent (BBR) in MABT) for 2-3 hours prior to overnight incubation

with a 1:1000 dilution of anti-Dig antibody (Roche), diluted in fresh blocking

buffer. This incubation was performed at 4^o^C.

The embryos were then washed 5 times for one hour in MABT and left

to wash overnight at 4oC. Embryos were then washed twice for 20 minutes at

room temperature in freshly made NTMT (100mM NaCl, 100mM TrisHCl pH

9.5, 50mM MgCl2, 0.1% TWEEN 20) and then stained with BCIP(175μg/ml)

and NBT(350g/ml) in NTMT. This reaction was carried out at 4oC in a light

proof container. The colour reaction was stopped with, and embryos stored

in, 4% formal saline at 4^o^C.

Before scanning under UV embryos require some further washes to

remove excess NBT/BCIP. Embryos were washed twice for 10 minutes in

PBS at RT and then moved to 10XTBST and allowed to equilibrate at RT, this

should take between 10-20 minutes depending on the size of the embryo.

Embryos were then washed 3 times for 20 minutes in 1XTBST and left to

wash overnight in fresh 1XTBST at 4^o^C. The following day embryos were

washed 3 times for 5 minutes in PBT at RT and then fixed overnight in

4%PFA –DEPC-PBS at 4°C. Embryos were washed briefly a further 2 times in

PBS and then refixed in Formal saline.

**OPT scanning:**

OPT scanning was carried out essentially following the protocol set out in

Sharpe et al. (Sharpe et al., 2002). The scan consists of 400 digital photos

taken through the embryo during a 360^o^ rotation, detecting and recording any

object interrupting the light path on the way. Standard reference embryos

were fixed in 4% PFA/0.2% glutaraldehyde mix, which produces a stronger

autofluorescent signal than PFA alone. This ensured a reasonable exposure

time for the photographs of the scan. The addition of 0.2% glutaraldehyde to

the fixative was not necessary for embryos that had been *in-situ* hybridised,

due to the presence of glutaraldehyde in the fixative steps of that protocol.

Reference embryos were stored in 100% methanol until scanning, at which

point they were taken back through a methanol series to PBS and briefly to

water. Embryos having undergone *in-situ* hybridisation were washed 3 times

for 20 minutes in PBS to remove storage fixative. In order to remove excess

salts embryos were washed twice for 10 minutes in distilled water and

subsequently left overnight in distilled water followed by 1 wash of 10 minutes

in fresh distilled water. Embryos were then embedded in 1% (w/v) low melting

point agarose and mounted with Permabond 200 onto a metal mount for

subsequent attachment to a magnet in the OPT scanner. Mounted embryos

were immersed in BABB (1:2 benzyl alcohol to benzyl benzoate) for at

least 4 hrs in order to clear the agarose and specimen. NBT stain can leach

out in this medium, and clearing should not be carried out for too long.

An autofluorescence scan of the sample is carried out first, followed by

a bright-field scan of the signal in the same sample. These operations take

<0.5hr. After scanning, the resulting tif images are processed using back

projection to convert the tif images into a 3-D digital volume in the Wlz data

format, the data is then cropped and converted to a 256 grey scale Wlz file.

**3D mapping:**

The mapping of the 3D gene expression data to the reference models was

performed using the Amira 4.1 software from Mercury Computer Systems.

Fluorescence and brightfield scans were imported into Amira and cropped to

the region of the forelimb. Fluorescent scans were then rendered as isosurfaces

and manually oriented in rough congruence to an isosurface for the

appropriate reference limb, The same positional transformations were then

applied to the brightfield signal data and both sets of data were duplicated to the same frame of reference as the reference limb.

Two corresponding sets of landmarks were set up between the

reference isosurface and the fluorescent scan isosurface. The landmarks were

based on prominent morphological landmarks such as the AER, the region

where the limb attaches to the flank and to proportional distances along the

main axes of the limb. In cases where there was a loss of anatomy due to

strong underlying signal either the greyscale data taken from a virtual section

of the fluorescent scan or a merged model of the brightfield and fluorescent

channels was used as a guide.

The fluorescent data was warped, using a Bookstein thin plate spline

method (Bookstein, 1989) provided by the Amira software and based on the

landmark sets. Provided the resulting warped fluorescent data seems

consistent with the reference limb’s morphology the same warp is then

applied to the brightfield channel data.

**Real-time PCR:**

Chick embryos (incubated for 4 days at 38^o^C) i.e., approx. stage 22-23, were

harvested in ice-cold PBS, the limb buds removed and the distal third cut off

with tungsten needles. These pieces were immediately transferred to

RNALater (Qiagen) in the smallest possible volume of PBS, samples of both limbs from 10 embryos were pooled in this way. A similar procedure was carried out on the proximal and median thirds. RNALater is designed to preserve the RNA in tissue samples for a considerable time.

RNA preps were made using the Qiagen RNA Easy micro kit, and checked

using an Agilent bioanalyzer, using their RNA 6000 nanochip. The integrity

values of all RNA samples were between 9.9 and 10 and the 28S and 18S

ratio between 1.9 and 2.1. These values indicate little degradation or

contamination.

500ng of each RNA sample were used to make cDNA. The reactions

consisted of 100ng oligo dT (30), plus distilled H2O to make the sample up to

10μl. These were then heated at 65^o^C for 3 mins and left to cool to room

temperature for 30 mins. To these the following were added: 50 U RNAsin,

10μg BSA; 1mM DTT, 0.5mM each dNTP, and 200 U MMLV reverse

transcriptase (Gibco-BRL). Reactions were incubated at 37^o^C for 1 hour.

Real-time PCR was carried out using an Applied BioSystems HT-7900

machine. Primers were selected from the Roche Universal Library using their

online software. For the *Wnt5a* reaction, probe 52 was used, and for *β-actin*,

probe 43 was used. Here the probes selected are not automatically checked

against other possible hybridisation targets in the chick, so this was carried

out manually by Blasting the candidate sequences against the chick genome

in Ensembl. The primers used for the *Wnt5a* reaction were: forward

5’catgatgaacctacacaatga 3’; reverse 5’ ccacgtcagccaggttgta 3’. And for the *β-*

*actin* reaction were: F 5’ cacacaagtgcccatttacga 3’; R 5’ caagtccagacgcaggatg

3’.

For the RT-PCR experiment itself, a standard curve for the *Wnt5a* RTPCR

was constructed by diluting a native plasmid containing the sequence

(Roslin ChEST no. 603799237F1) to give DNA concentrations of 106

molecules per reaction down to 100 molecules in a 10-fold dilution series. A

standard curve for the *β-actin* quantitation control was constructed using a

cDNA preparation from whole stage 21 chick embryos. Here RT-PCR

reactions contained a volume of the initial cDNA reaction equivalent to an

input of 10ng of RNA per reaction down to 0.001ng per reaction in a 10-fold

dilution series. Similar dilution series were made from the proximal, medial

and distal limb bud cDNA preparations for both *Wnt5a* and *β-actin* PCR

reactions. PCR reactions were carried out in triplicate. The results from the

PCR reactions were analysed statistically using the sDS.1 package with

Excel.

**Computational analysis:**

The AMIRA arithmetic module was used for a number of simple computational procedures such as deriving average intensity values based on a set of expression data sets. This package was also used to derive common domains of expression between data sets by masking voxels where in any one data set the intensity value of those voxels was zero, this left only voxels which were non-zero in all data sets.

To derive mean values for grey level intensity representing gene expression within a domain, as in Fig.1.F, the Amira software package segmentation editor was used to define distinct domains of the limb. These domains were then used as queryable regions within Amira for measuring the mean grey level intensity.

To derive the mean values along the proximo-distal axis, Fig.1.D, both the brightfield and the fluorescent data sets were resectioned along the proximo distal axis. The MRC HGU developed software package MAPaint was used to orient a sectioning plane to the P-D axis and the orientation saved in the “.bib” file format. This file and the original data sets were then used as inputs for the Wlz3DGetSection program which produced a series of images in the Wlz format representing sections along the proximo-distal axis. These were then converted into a 3D data file using the [WlzConstruct3D](ftp://ftp.hgu.mrc.ac.uk/pub/MouseAtlas/bin/Solaris/WlzConstruct3D) program and then to a tiff stack using the WlzExtFFConvert program. The tiff stacks were then imported into the ImageJ image processing software package. The fluorescent data was thresholded to remove background and used to mask the brightfield data using the AND arithmetical operation. The masked brightfield dataset was then thresholded and the ‘Plot Z-axis profile’ command used to measure the mean grey level intensity in each slice.

To derive the median values of several data sets we used a Wlz software tool developed by the MRC, WlzFilterNObjsValues which ranks the values for voxels at the same position in different data sets and produces a new data set composed of the median values for every voxel. This tool is available from the MRC HGU FTP site at <ftp://ftp.hgu.mrc.ac.uk/pub/MouseAtlas/bin/> in formats suitable for Mac, PC and Unix based systems.

For more complex computational analyses each of the experimental gene-expression spatial distributions has been mapped into the standard coordinate framework defined by the model limb. To analyse the gene-expression patterns we first divide the limb into 560 non-overlapping sub-regions of 10x10x10 voxels each. Each of these is used to sample the experimental gene-expression patterns. For each experimental pattern the mean gene-expression strength (integrated optical density divided by the volume) within each box is calculated. If the box is partially external to the limb then only the intersecting volume is considered. By this means a 2D matrix of mean expression strengths across the limb was calculated. Each row of the matrix for a given gene is a low-resolution representation of the pattern and each column for a given sample-region is the genetic “signature” for that spatial location.

The resulting matrix of gene expression values was analysed using the

TMEV4 package from TIGR. The data were analysed using a hierarchical

clustering method to produce a nested tree of gene expression pattern

similarity based on a Euclidean distance metric. A nested tree was also

produced of the similarity of domains of the 3D data model based on gene

expression. The resulting tree was then used to identify clusters made of

small groups of regions at the terminus of long branches. These regions were

used to produce larger 3D domains corresponding to the whole volume

occupied by the regions comprising each cluster, which could be

subsequently visualised. Visualisation was performed using the Amira

software package.

**References:**

Abramoff, M.D., Magelhaes, P.J., Ram, S.J., (2004), Image Processing with

ImageJ. *Biophotonics International* **11**, issue 7, 36-42.

Bookstein, F. (1989). Principal warps: Thin-plate splines and the

decomposition of deformations. *IEEE Transactions on Pattern Analysis and MachineIntelligence* **11,** 567-585.

Crossley, P. H., Minowada, G., MacArthur, C. A., and Martin, G. R. (1996).

Roles for FGF8 in the induction, initiation, and maintenance of chick limb development. *Cell* **84,** 127-36.

Echelard, Y., Epstein, D. J., St-Jacques, B., Shen, L., Mohler, J., McMahon, J.

A., and McMahon, A. P. (1993). Sonic hedgehog, a member of a family of putative signalling molecules, is implicated in the regulation of CNS polarity. *Cell* **75,**1417-30.

Hill, R. E., Jones, P. F., Rees, A. R., Sime, C. M., Justice, M. J., Copeland, N.

G.,Jenkins, N. A., Graham, E., and Davidson, D. R. (1989). A new family of mouse homeo box-containing genes: molecular structure, chromosomal location, and developmental expression of Hox-7.1. *Genes Dev* **3,** 26-37.

Isaac, A., Rodriguez-Esteban, C., Ryan, A., Altabef, M., Tsukui, T., Patel, K.,

Tickle, C., and Izpisua-Belmonte, J. C. (1998). Tbx genes and limb identity in chick Embryo development. *Development* **125,** 1867-75.

Maniatis, T., Fritsch, E., and Sambroook, J. (1982). "Molecular Cloning: A

Laboratory manual " Cold Spring Harbor Laboratory Press, New York.

Nieto, M. A., Patel, K., and Wilkinson, D. G. (1996). In situ hybridization analysis of chick embryos in whole mount and tissue sections. *Methods Cell Biol* **51,** 219-35.

Sharpe, J., Ahlgren, U., Perry, P., Hill, B., Ross, A., Hecksher-Sorensen, J.,

Baldock, R., and Davidson, D. (2002). Optical projection tomography as a tool for 3D microscopy and gene expression studies. *Science* **296,** 541-5.
